# A lab-on-chip for malaria diagnosis and surveillance

**DOI:** 10.1186/1475-2875-13-179

**Published:** 2014-05-09

**Authors:** Brian J Taylor, Anita Howell, Kimberly A Martin, Dammika P Manage, Walter Gordy, Stephanie D Campbell, Samantha Lam, Albert Jin, Spencer D Polley, Roshini A Samuel, Alexey Atrazhev, Alex J Stickel, Josephine Birungi, Anthony K Mbonye, Linda M Pilarski, Jason P Acker, Stephanie K Yanow

**Affiliations:** 1 School of Public Health, University of Alberta, WMC 2B4.59, 8440 – 112th Street, Edmonton, AB, Canada; 2 Provincial Laboratory for Public Health, Edmonton, AB, Canada; 3 Aquila Diagnostic Systems Inc, Edmonton, AB, Canada; 4 Department of Oncology, University of Alberta, Edmonton, AB, Canada; 5 Department of Clinical Parasitology, Hospital for Tropical Diseases, London, UK; 6 Uganda Virus Research Institute, Entebbe, Uganda; 7 School of Public Health, Makerere University, Kampala, Uganda; 8 Ministry of Health, Kampala, Uganda; 9 Canadian Blood Services, Edmonton, AB, Canada

## Abstract

**Background:**

Access to timely and accurate diagnostic tests has a significant impact in the management of diseases of global concern such as malaria. While molecular diagnostics satisfy this need effectively in developed countries, barriers in technology, reagent storage, cost and expertise have hampered the introduction of these methods in developing countries. In this study a simple, lab-on-chip PCR diagnostic was created for malaria that overcomes these challenges.

**Methods:**

The platform consists of a disposable plastic chip and a low-cost, portable, real-time PCR machine. The chip contains a desiccated hydrogel with reagents needed for *Plasmodium* specific PCR. Chips can be stored at room temperature and used on demand by rehydrating the gel with unprocessed blood, avoiding the need for sample preparation. These chips were run on a custom-built instrument containing a Peltier element for thermal cycling and a laser/camera setup for amplicon detection.

**Results:**

This diagnostic was capable of detecting all *Plasmodium* species with a limit of detection for *Plasmodium falciparum* of 2 parasites/μL of blood. This exceeds the sensitivity of microscopy, the current standard for diagnosis in the field, by ten to fifty-fold. In a blind panel of 188 patient samples from a hyper-endemic region of malaria transmission in Uganda, the diagnostic had high sensitivity (97.4%) and specificity (93.8%) versus conventional real-time PCR. The test also distinguished the two most prevalent malaria species in mixed infections, *P. falciparum* and *Plasmodium vivax*. A second blind panel of 38 patient samples was tested on a streamlined instrument with LED-based excitation, achieving a sensitivity of 96.7% and a specificity of 100%.

**Conclusions:**

These results describe the development of a lab-on-chip PCR diagnostic from initial concept to ready-for-manufacture design. This platform will be useful in front-line malaria diagnosis, elimination programmes, and clinical trials. Furthermore, test chips can be adapted to detect other pathogens for a differential diagnosis in the field. The flexibility, reliability, and robustness of this technology hold much promise for its use as a novel molecular diagnostic platform in developing countries.

## Background

Malaria is a blood-borne parasitic disease causing over 660,000 deaths each year, primarily in young children from sub-Saharan Africa [1]. In the past decade, malaria control efforts have focused on scale-up interventions including use of insecticide treated bed nets, indoor residual spraying, rapid diagnostic tests and artemisinin-based combination therapy to reduce morbidity and mortality. Still, in countries bearing the largest malaria burden, access to quality diagnostics is limited [2,3]. Microscopy, if available, is the gold standard for malaria diagnosis but in health centres with limited resources, malaria is diagnosed presumptively in patients with fever [4]. These practices lead to overtreatment and misdiagnosis of potentially fatal bacterial infections and are becoming less feasible in the face of emerging drug resistance and costly new therapies [2,5,6]. As such, new diagnostic practices are urgently needed. Furthermore, as malaria control efforts intensify and countries progress to elimination phases, sensitive diagnostics will play a key role in surveillance to identify sporadic outbreaks and asymptomatic reservoirs of infection [7]. Immunochromatographic rapid diagnostic tests (RDTs) provide an important alternative to microscopy at the point-of-care, but are not without significant limitations, including decreased sensitivity at lower levels of parasitaemia, inhibition at high levels of parasitaemia (prozone effect), inability to quantitate or distinguish malaria species in mixed infections, and failure to detect parasites with mutations in the genes encoding certain target antigens [4,8,9]. While strides have been made in improving RDT performance [10], nucleic acid based diagnostics, including PCR and isothermal amplification, provide superior sensitivity and specificity [11]. However, the need for pre-analytic processing, cold storage, and expensive equipment has precluded the use of nucleic acid diagnostics in the field. Lab-on-chip platforms have shown promise in overcoming these barriers by adapting common laboratory tests to a self-contained, portable, micro-scale format targeted to the point-of-care [12]. In this study, the development of a plastic hydrogel chip and a portable real-time PCR machine for malaria diagnosis is described.

## Methods

### Patient samples and controls

Archived, frozen samples from malaria patients with a positive diagnosis by PCR were acquired from the Provincial Laboratory for Public Health (ProvLab) in Edmonton, Alberta. The blind panel run on the Gelcycler was generated from samples collected in 2011–2012 in Uganda from consenting pregnant women participating in a study to genotype parasite resistance [13] (during antenatal care, at delivery and those with fever attending outpatient clinics). Blood (4 mL) was sampled by venipuncture, stored in EDTA vacutainers, and transferred to the Uganda Virus Research Institute for storage at −20°C. Thick smears were stained by Giemsa and malaria parasites counted against 200 leukocytes and expressed as number of parasites per μL of blood assuming a standard leukocyte count of 8,000/μL of blood. A smear was considered negative after examining a minimum of 100 high power fields with no parasites seen. Microscopy was performed by a laboratory technician at one of two health facilities in Mukono district. Coded samples were aliquoted and shipped frozen for testing in Alberta. Ethical approval was granted by the review boards at the Uganda Virus Research Institute and Uganda National Council for Science and Technology (Reference HS 747). Use of samples from ProvLab and the Ugandan study was approved by the Health Research Ethics Board at the University of Alberta.

The blind panel run on the Accutas comprised randomly chosen EDTA samples as positive and negative for malaria DNA that were submitted during the first seven months of 2011 for routine malaria diagnosis at the Department of Clinical Parasitology, Hospital for Tropical Diseases [14].

A nested PCR reaction [15] to amplify malarial DNA was performed on all these samples in addition to routine diagnosis by microscopy. All PCR positive samples were further characterized against the WHO International Standard for *Plasmodium falciparum* by a real-time PCR reaction [16]. Once a definitive malaria diagnosis had been made for each sample the remainder were anonymized and stored as surplus to diagnostic requirements for use in ethically approved research projects. The panel consisted of positive samples infected with the following species: *P. falciparum* (n = 25), *Plasmodium vivax* (n = 2), *Plasmodium ovale* (n = 2), and *Plasmodium malariae* (n = 1). Parasitemia ranged from <5 to >1,000 parasites/μL blood. The panel also included eight negative samples.

*Plasmodium knowlesi* purified genomic DNA (Malaria Research and Reference Reagent Resource Center (MR4), Manassas, VA) was diluted to 2 ng/mL in non-infected blood to serve as a reference for this species. The negative control used in this study was a hemoglobin tri-level control, level 2 (Stanbio, Boerne, TX) diluted 1:10 in water. The positive control had the same composition as the negative, with the addition of a consensus PCR product to a final concentration of 10^5^ copies/μL for hydrogel wax chips and 10^2^ copies/μL for hydrogel plastic chips.

### Parasite culture

*Plasmodium falciparum* parasites were grown in human erythrocytes at 3-5% haematocrit as described [17]. Ring stage parasites were enriched by sorbitol lysis [18], counted, and diluted in whole blood. The diluted parasites were frozen until use.

### Reagents

The hydrogel mastermix contains reagents described previously for direct amplification of DNA targets from blood [19] and reagents for forming a polyacrylamide gel. This mastermix was prepared with 1X Klentaq Buffer (containing 3.5 mM Mg^2+^; DNA Polymerase Technology, St. Louis, Mo), 40X SYBR Green I (Life Technologies, Grand Island, NY), 1X PCR Enhancer Cocktail (PEC-1) (DNA Polymerase Technology), 200 nM forward and reverse primers, 200 μM dNTPs, 0.03% BSA, 4% acrylamide (Sigma St. Louis, Mo), 0.4% bis-acrylamide aqueous solution (BioRad, Hercules, CA), 0.06% azobis (Wako, Richmond, VA), and 0.1% TEMED (Sigma). For a 100 μL mastermix volume, 3 μL of Omni KlenTaq enzyme was added (DNA Polymerase Technology). Both consensus PCR primers [15,20], and species-specific primers [21,22] target regions of the 18S rRNA gene of *Plasmodium*.

### Hydrogel wax chips

A prototype consisting of a wax cassette in a 23.5 × 32 mm aluminum pan was created wherein the hydrogel is polymerized and desiccated on a coverslip above a wax-bound “trench”. A polydimethylsiloxane (PDMS) stamp was used to imprint 4 trenches in the wax. Three coverslips (20 mm × 5 mm) were placed equally spaced on top of the wax, perpendicular to the trenches, creating 12 covered slots for hydrogels. Coverslips were sealed onto the wax by briefly heating the surface of the coverslip between each trench with a soldering iron, allowing the wax underneath to melt slightly. A 13 μL aliquot of mastermix was added to each slot, creating a “gel strip”. The gel strip was photopolymerized at room temperature under UV light (367 nm) for 30 minutes. Polymerized gels were desiccated under vacuum (23 inHg) for 1 hour at room temperature and used within seven days. Chips were stored in dark conditions to prevent photo-bleaching of SYBR green dye. Coverslips were treated with 3-(trimethoxysilyl)propyl methacrylate to promote adherence to the gel strip as described [23]. On desiccation, the gel shrinks away from the trench and stays attached to the coverslip, creating a channel underneath the strip where the sample can diffuse in. Whole blood was diluted 1:10 with water, then 8.5 μL of this sample mixture was applied underneath the coverslip/gel-strip. After the addition of samples and controls, the wax cassette was incubated for 10 minutes at room temperature then placed directly on the Peltier element within the Gelcycler for thermocycling.

### Hydrogel plastic chips

Plastic chips were manufactured by miniFAB (Scoresby, Australia) by injection molding of cyclic olefin polymer (COP1420R) into top (27 × 27 mm) and bottom pieces (27 × 42 mm). To form the hydrogels, a 15 μL aliquot of mastermix was first added to the wells in the top piece. Next, the top piece was placed in a customized UV illumination chamber and flooded with nitrogen for 2 minutes. The gel was photopolymerized at room temperature under UV light (405 nm) for 30 minutes. The chamber was flooded with nitrogen a second time for 2 minutes, halfway into the UV treatment. This sustained nitrogen overlay was needed to prevent oxygen from inhibiting gel polymerization. The polymerized gels were desiccated under vacuum (23 inHg) at least 20 hours at room temperature. To complete the process of making a chip, a 10 μL aliquot of wax was added to each reservoir in the bottom piece, and the top and bottom pieces were fitted together. Wax in the bottom reservoir melts during thermocycling to cover the gel and sample ports, preventing evaporation and further cross-contamination that may occur at high denaturation temperatures. A two-sided tape adhesive on the bottom piece seals the chip upon assembly and prevents cross-contamination between gel chambers. The assembled plastic chips were used within 24 hours of preparation, or vacuum-sealed in plastic, stored at room temperature (22°C) in the dark, and tested within one month. With this design, sample added via the sample port in the top piece enters the lower reservoir and rehydrates the gel in the well. However, to ensure efficient loading of the sample in the validation experiments presented here, 8.5 μL of whole blood diluted 1:10 in water was added directly to the gel in the well before assembly of the chip. Samples were incubated prior to thermocycling as described above. As opposed to the wax chips, which sit freely on the Gelcycler Peltier element, the plastic chips were secured onto the Peltier with a gantry to ensure evenly distributed thermal transfer.

### Instrumentation: Gelcycler and Accutas

The Gelcycler features a Peltier element for sample heating, components for stringent thermal regulation, a laser and CCD camera for capturing fluorescence [24]. Consensus PCR reactions for the hydrogel wax chip were performed with a 10 minute initial denaturation step at 94°C, followed by 40 cycles of 94°C for 20 seconds, 58°C for 30 seconds, 72°C for 30 seconds, and a 2 minute final extension of 72°C. Reaction conditions for the hydrogel plastic chips were the same as above, except the consensus PCR annealing temperature was 60°C, and the species-specific annealing temperature was 64°C. MCA from 70-90°C was performed immediately after PCR. Fluorescent images were obtained and analysed as described [24] to generate real-time PCR and MCA curves using in-house software that requires manual analysis. A five-parameter sigmoid curve-fitting method using the first derivative was created to calculate cycle threshold (Ct) values. Samples were considered positive if they had a Ct value of less than 40 and the expected MCA peak profile. Conventional real-time PCR confirmation was performed on panel samples as described in Taylor et al. [19]. The Accutas system refines the Gelcycler and brings many improvements. The modular design incorporates Design for Manufacturing principles to enable low cost, high volume manufacturing of the system. The Accutas system includes an LED light source rather than a laser-based system, allowing lower power and safer operation. The temperature-controlled environment allows the Accutas to be run in a wider window of temperatures. The temperature control of the Accutas was optimized and is easily adaptable to new designs. The new aluminum framed enclosure is more rugged for transport in the field. The PC control software was rewritten as well to provide an easier, step-by-step user interface with automated reporting at the end of each experiment.

### Statistics

Analyses were performed using SAS/STAT 9.1. Confidence intervals (95%) were calculated using the Wilson score interval method.

## Results

### Test performance in prototype hydrogel chips

**Figure 1:**
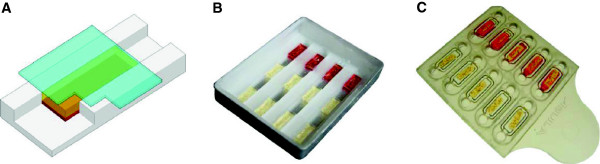
**A prototype hydrogel wax chip and manufactured plastic chip for malaria diagnosis. (A)** Core concept for hydrogel PCR. A single hydrogel strip (yellow) is desiccated and adheres to one surface of the chip creating a channel underneath for sample delivery. Blood flows through the channel by capillary force and is absorbed by the gel. **(B)** A wax chip with trenches overlaid with hydrogel bound to a glass coverslip. Blood is manually loaded into the channel below each hydrogel strip. **(C)** A plastic chip composed of top and bottom pieces of molded plastic. The gel is housed within a well in the top piece. In the current study, blood was added directly to the hydrogel in the open chip, and the two pieces of plastic were sealed prior to PCR. With this design, blood can also be added via the circular sample ports in the pre-sealed chip.

In previous studies “hydrogel” PCR technology was developed to package PCR reagents in a format that can be stored and is easy to use [23]. This included the creation of a customized field-ready real-time PCR machine, the ‘Gelcycler’. In a separate study, a conventional PCR method for detecting malaria directly from blood samples, without the need for prior DNA purification, was developed [19]. The goal was to translate this research into a disposable lab-on-chip diagnostic that would find practical application in field settings. The initial phase of this work focused on creating a prototype chip to which multiple blood samples could be applied and tested. These prototype chips were created in the lab entirely from readily available materials. Aluminum cosmetic pans served as the base of the chip; these were filled with molten wax and four separate troughs were created using a stamp [24] (Figure 1A and 1B). Once the stamp was removed, glass coverslips cut to the appropriate size were laid over the troughs in the hardened wax, generating discrete channels to contain the hydrogels. The hydrogel mastermix including all of the reagents necessary for real-time PCR from blood was added to each channel underneath the coverslip. The mastermix was polymerized and desiccated, yielding 12 self-contained hydrogel units intended for 12 blood samples to be tested in the PCR reaction [24,25]. During desiccation, the hydrogel shrinks away from the wax trough but stays attached to the coverslip, creating a route for sample delivery via the channel underneath each gel (Figure 1A). Addition of blood to the chip rehydrates the gel within minutes and the blood is retained within the gel unit. During PCR, the wax melts and surrounds the hydrogels attached to the coverslip. The hydrogels remain attached in their original positions during PCR, and no cross-contamination was observed with this chip configuration.

**Figure 2:**
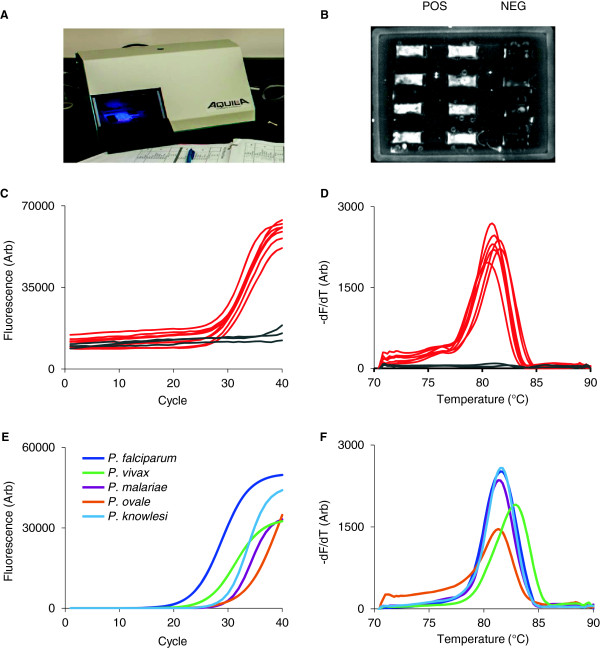
**Malaria detection on hydrogel wax chips. (A)** The ‘Gelcycler’, a custom-built, miniaturized real-time PCR instrument. **(B)** Raw SYBR green fluorescence images from positive and negative control wells in the wax chip. **(C)** Real-time PCR amplification curves and **(D)** melt curve analysis of the negative (gray) and positive (red) controls in **(B)**. **(E, F)***Plasmodium* DNA detected from clinical samples and a spiked *P. knowlesi* sample using PCR with conserved primers. Positive samples exhibit an amplification curve **(E)** and melt curve **(F)** at the appropriate melting temperature for each species.

Prototype chips were run on the Gelcycler which performs real-time PCR followed by melt curve analysis (MCA) to confirm the specificity of the PCR product (Figure 2A). In this reaction, consensus primers target a region of the 18*S* rRNA gene from *Plasmodium*[19] and amplified DNA is detected by SYBR green chemistry (Figure 2B). Real-time PCR (Figure 2C) and MCA curves (Figure 2D) using positive and negative controls demonstrated high reproducibility. Using clinical samples from patients with imported malaria in Canada, the consensus reaction successfully amplified five species of *Plasmodium* directly from blood (Figures 2E and 2F). The limit of detection for *P. falciparum* was 2 parasites/μL, measured in serial dilutions of cultured parasites in whole blood (Additional file 1).

### Manufactured plastic chips for hydrogel PCR

In the next phase of chip development, an enclosed plastic chip was designed that retained the key features of the prototype wax chip and could be manufactured on a large scale for use in the field (Figure 1C). The thermal properties and auto-fluorescence of several types of plastics were assessed and cyclic olefin polymer (COP1420R) proved most compatible with real-time PCR. The top piece of the chip was molded with 10 individual wells: eight test wells and two controls. Two ports for sample delivery are present on either side of each well. The hydrogel mixture was added to each well, polymerized and desiccated. A bottom piece of plastic was created with reservoirs that align with the sample ports and wells of the top piece. When the two pieces are adhered, they form an enclosed chip with channels for sample delivery, similar in concept to the hydrogel wax chip. The assembled plastic chips are easily packaged and stored. Blood samples were simply diluted in water for lysis (2 μL of blood added to 18 μL of water) and 8.5 μL was added to the hydrogel on the chip. This corresponds to approximately 0.85 μL of the original blood sample. To ensure accuracy in the current study, samples were added to each hydrogel prior to chip assembly. Positive and negative controls were included in each chip to ensure on-board quality control for the reagents and amplification reactions (Additional file 2).

### Test performance in plastic chips

**Figure 3:**
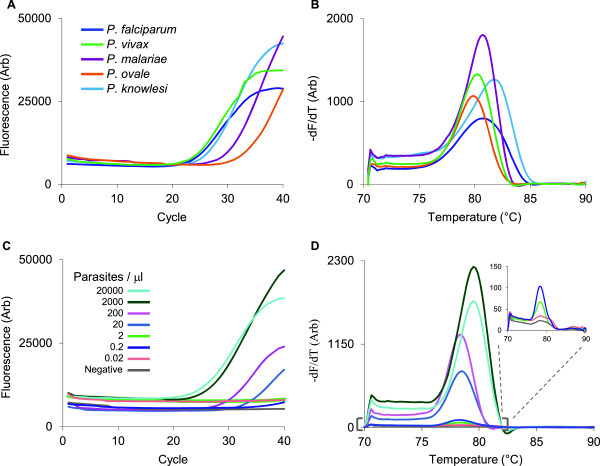
**Diagnosis in hydrogel plastic chips. (A, B)** On-chip detection of parasite DNA directly from clinical samples and a spiked *P. knowlesi* sample with the consensus PCR primers. **(C, D)** Limit of detection analysis with dilutions of ring-synchronized *P. falciparum* parasites grown in culture.

Compared with our prototype wax chip, the hydrogel plastic chip showed similar, if not superior performance of species coverage (Figure 3A and 3B) and limit of detection (2 parasites/μL; Figure 3C and 3D). This sensitivity corresponds to 1.7 parasites per 0.85 μL of blood sampled in each well. With approximately seven copies of the target gene per parasite genome [26], this equates to 12 gene copies detected. The plastic chip demonstrated excellent performance within a range of 2 × 10^5^ to 0.2 parasites/μL, consistent with parasite levels observed in the field.

**Figure 4:**
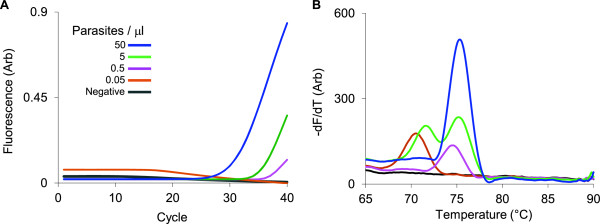
**PCR sensitivity is maintained after storage. (A, B)** Serial dilutions of *P. falciparum* cultures run on chips that were loaded with hydrogel reagents, desiccated and stored for 4 months at 37°C.

To assess the stability of the reagents at high temperatures, chips were loaded with hydrogel reagents, desiccated, sealed, and stored for 4 months at 37°C (Figure 4A and 4B). The sensitivity of the PCR was maintained after storage with detection at concentrations of 0.5 parasites/μL.

### Validation of plastic chips

The plastic chips were further validated using a blind panel of 188 samples from pregnant women residing in a hyper-endemic region in Uganda. Women were either attending antenatal clinics (98%) or in delivery (2%) at the time of blood collection and most presented with fever (Table 1). Based on microscopy performed at the local hospital in Uganda, 142 samples were positive with a median parasitaemia of 440 parasites/μL. The chip had very high sensitivity compared with microscopy (96.5%; Table 2). Specificity was low (63.0%), which was expected given that the analytical sensitivity of the chip is at least one order of magnitude greater than microscopy. Importantly, the chip detected 17 positive samples that were negative by microscopy; 13 were from asymptomatic patients and four with acute malaria. For comparison, and to resolve discordant results, the panel was also tested on a conventional real-time PCR platform using the direct from blood assay [19]. The chip had a sensitivity of 97.4% and specificity of 93.8% compared with conventional real-time PCR. The four samples that were positive by conventional real-time PCR but negative on the chip had cycle threshold (Ct) values >38, suggesting they were very low level infections and perhaps undetected due to sampling error. Overall concordance between the two PCR methods was 96.8% with a kappa statistic of 0.890.

**Table 1:** Demographic and clinical characteristics of patients included in the blind panel from Uganda (n = 188)

**Median age [range]**	**20 years [14 – 42]**
Presentation	
Asymptomatic (n [%])	72 [38.3]
Clinical malaria (n [%])	116 [61.7]
Pregnancy status	
In antenatal care (n [%])	184 [97.9]
At delivery (n [%])	4 [2.1]
Median parasitemia [range]	440 parasites/μL [1 – 94800]

**Table 2:** Performance characteristics of the hydrogel plastic chip compared with microscopy and conventional real-time PCR

		**Chip PCR**				
		**Pos (n)**	**Neg (n)**	Sensitivity* (95% CI)	Specificity* (95% CI)	**Kappa (95% CI)**
**Microscopy**	Pos (n)	137	5	96.5% (92.0 – 98.5)	63.0% (48.6 – 75.5)	0.653 (0.521 – 0.785)
	Neg (n)	17	29			
**Conventional real-time PCR**	Pos (n)	152	4	97.4% (93.5 – 99.0)	93.8% (79.9 – 98.3)	0.890 (0.803 – 0.976)
	Neg (n)	2	30			

*Determined using microscopy or conventional real-time PCR as the comparator.

### Malaria speciation on plastic chips

**Figure 5:**
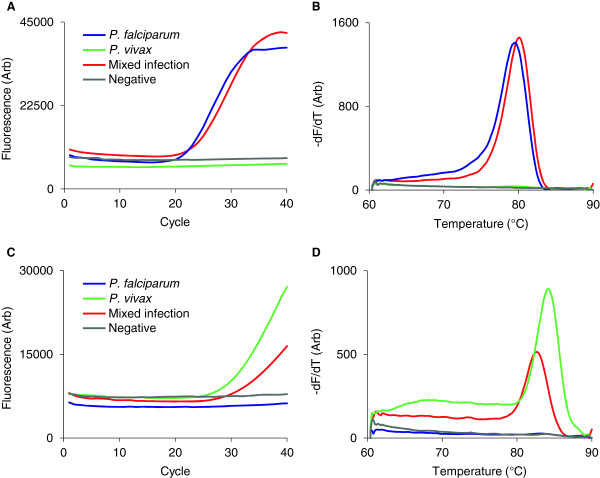
**On-chip detection of *P. falciparum *and *P. vivax*.** Clinical samples from patients infected with *P. falciparum* (blue) and *P. vivax* (green), a 1:1 mixture of each species (red) or uninfected (gray), were tested on hydrogel plastic chips with PCR primers specific for *P. falciparum***(A, B)** and *P. vivax***(C, D)**. Amplification (left panels) for each species was confirmed by melt curve analysis (right panels).

In many malaria-endemic regions, multiple species of *Plasmodium* co-exist which require specific treatment regimens and targeted control measures. The consensus PCR is unable to discriminate between species based on melt curve analysis (Figure 3B) and primer competition would prevent detection of mixed infections. Species-specific primers were designed that amplify the two major species of *Plasmodium*, *P. falciparum* and *P. vivax*, from the same sample but in separate wells on the chip. Following testing for cross-reactivity, one primer set for each species was identified that specifically amplifies *P. falciparum* (Figure 5A and 5B) and *P. vivax* (Figure 5C and 5D) from single infections and a 1:1 mixture of both species. Addition of these reactions to the chip expands its functionality from genus to species-level diagnosis.

### Development and validation of a field-ready device

**Figure 6:**
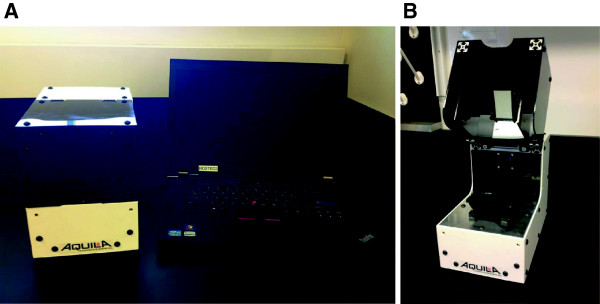
**The ‘Accutas’ system**. Shown with a laptop in **(A)** and with the top open **(B)**, this smaller, rugged design features an LED light source, operability within a wider ambient temperature range, and automated data reporting.

Once proof-of-concept was demonstrated on the Gelcycler prototype, a new instrument that streamlines manufacturing and usability in the field, called the ‘Accutas’ , was designed. This device (Figure 6) includes several improvements over the Gelcycler: an LED light source instead of a laser for fluorescence detection, temperature-controlled environment, framed enclosure, and software with a simple user interface. To assess the performance of the Accutas, a third-party evaluation with a blind panel of 38 samples from the Hospital for Tropical Diseases in London was conducted. Samples were collected from patients with imported malaria, including four species of *Plasmodium*, and negatives (Table 3). Of 30 positive samples, 29 were detected on the Accutas, yielding a sensitivity of 96.7%. The specificity was 100%. This third party evaluation was completed before the speciation reaction had been validated on the Gelcycler.

**Table 3:** Third party assessment of the Accutas (n = 38)

		**Chip PCR**				
		**Pos (n)**	**Neg (n)**	**Sensitivity (95% CI)**	**Specificity (95% CI)**	**Kappa (95% CI)**
**Microscopy**	Pos (n)	29	1	96.7% (83.3 – 99.4)	100% (67.6 – 100.0)	0.924 (0.778 – 1.000)
	Neg (n)	0	8			

## Discussion

Accessible, quality diagnostics with low infrastructure requirements are expected to profoundly impact public health and child mortality from malaria [3]. They also support new ‘test-before-treat’ policies to curb the emergence of drug-resistant parasites and discourage presumptive diagnosis based on fever [27,28]. Highly sensitive molecular technologies exist with the potential to meet this critical need [8,11], yet few have overcome the barriers of sample preparation, cold storage, cost, complexity and infrastructure requirements for use in low-resource settings [29]. The recent clinical evaluation of a commercial loop-mediated isothermal amplification (LAMP) kit that amplifies parasite DNA with minimal extraction steps addresses many of these issues and demonstrates that molecular testing in the field is feasible [14,30,31]. However, while the innovations present in field-ready LAMP kits are commendable, a separate sample preparation process is still a necessary part of the procedure. This step, even in streamlined self-contained form provided by commercial kits, can be a significant barrier to implementation of a diagnostic test in resource-limited areas.

In this work a PCR-based diagnostic for malaria intended for use at or near the point-of-care is presented. The platform consists of hydrogel chips run on a customized portable real-time PCR machine. The hydrogel chips are disposable, do not require a cold-chain, and can be stored and used on demand for a result in less than two hours. PCR performed directly with unprocessed blood eliminates a major obstacle to adopting this technology outside of reference or research labs, and simplifies the testing for unskilled users. The entire system was designed to be low-cost for use in developing countries. The chips use small volumes of master mix (15 μL per test), and the material cost for the chips will be further reduced to under $1 USD per test with scaled production. Through innovative engineering and with mass production, the real-time PCR instrument can be manufactured for less than $2,000 USD. Quality optics constitutes the major share of component costs, but is essential to maintain sensitive fluorescence detection. The instrument is designed to operate on 12 V which can be supplied by a variety of power sources include the grid, dry cells, generators or photovoltaic systems. The amount of power required to run the device is equivalent to a car battery, which can be used to power the device in field settings where the supply of electricity is unstable. In the next generation of the platform, direct loading of the chip and a battery-operated instrument that provides a result within an hour will fully adapt the technology to the constraints of the field.

## Conclusions

The technology described in this study has the potential to make major inroads by supporting key areas of malaria control: as a diagnostic for acute malaria, for surveillance in elimination settings, and as a tool in clinical evaluations of new drugs and vaccines. Importantly, there are numerous applications of this technology beyond malaria. The platform has the inherent flexibility to detect any molecular target simply by using different PCR primers in the hydrogel mix. This marks an important advantage over other approaches, such as LAMP, that require extensive primer validation. Tests for other disease targets in different specimen types are readily adapted to our platform, for example, sexually transmitted infections (STI) detected from urine and genital swabs [24]. Furthermore, as shown here with malaria species, and separately for STI targets [24], a single patient sample could be tested for multiple targets simultaneously, providing a rapid differential diagnosis. Chips can be tailored to test for different pathogens, species, or genetic markers (including drug resistance markers), depending on the clinical or public health need. In summary, this work showcases a novel molecular technology that can make an important contribution to the diagnosis and surveillance of infectious diseases in low-resource settings.

## Competing interests

AH, SDC, DPM, WG, AA, AJS and JPA have competing interests or other interests that might be perceived to influence the results and/or discussion reported in this article. AH, SDC and WG are employees of Aquila Diagnostic Systems Inc. AJS is a shareholder of Aquila Diagnostic Systems Inc. AA, DPM, and JPA hold pending patents related to the technology described in this manuscript. JPA is a founder and shareholder of Aquila Diagnostic Systems Inc.

## Authors’ contributions

BJT, KAM, and RAS optimized hydrogel PCR using blood. AH and SDC optimized wax and plastic chip PCR. AH performed chip PCR on the panel from Uganda and SP performed chip PCR on the panel from HTD. BJT, SL and AJ optimized species-specific PCR on the chips. KAM and AH conducted conventional real-time PCR and KAM maintained parasite cultures. DPM conceived the hydrogel wax chips and AJS designed the hydrogel plastic chips. DPM developed hydrogel desiccation and sample delivery with contributions from RAS. AA conceived hydrogel PCR and tested sample delivery methods. AJS and WG designed, assembled and programmed the Gelcycler. AM and JB supervised collection of blood samples and clinical data. JPA and LMP supervised the chip and device development arm of the study. SKY conceived the study and supervised the diagnostic arm of the study. SKY and BJT wrote the manuscript. All authors read and approved the final manuscript.

## Supplementary Material

Additional file 1 **Limit of detection analysis on hydrogel wax chips.** (A) PCR and (B) MCA performed using dilutions of ring-synchronized *P. falciparum* parasites grown in culture and serially diluted in whole blood. Click here for file

Additional file 2 **Hydrogel plastic chip controls.** (A) PCR and (B) MCA curves for negative (gray) and positive (red) controls. Click here for file
