# Supplementary material for: A lab-on-chip for malaria diagnosis and surveillance
Source: Malar J. 2014 May 9;13:179. doi: 10.1186/1475-2875-13-179 (PMC4029813; doi:10.1186/1475-2875-13-179)
Supplement: Additional file 1 — Limit of detection analysis on hydrogel wax chips. (A) PCR and (B) MCA performed using dilutions of ring-synchronized P. falciparum parasites grown in culture and serially diluted in whole blood. [file 1475-2875-13-179-S1.pptx]

## Slide 1
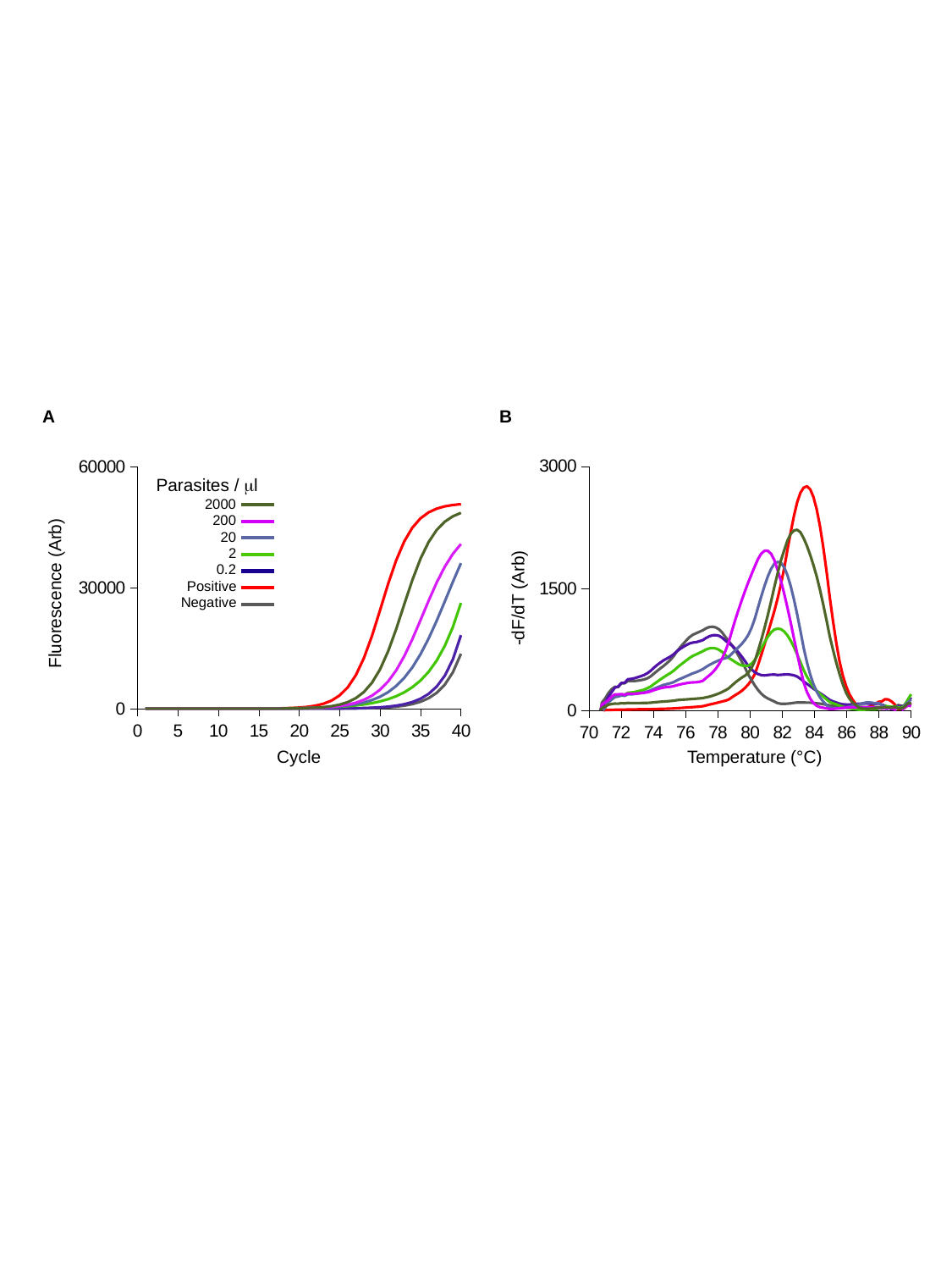

A
B
### Chart
| Category | | | | | | | |
|---|---|---|---|---|---|---|---|
### Chart
| Category | | | | | | | |
|---|---|---|---|---|---|---|---|Parasites / ml
2000
200
20
2
0.2
Positive
Fluorescence (Arb)
-dF/dT (Arb)
Negative
Cycle
Temperature (°C)
