# Supplementary figures and images for: A lab-on-chip for malaria diagnosis and surveillance
Source: Malar J. 2014 May 9;13:179. doi: 10.1186/1475-2875-13-179 (PMC4029813; doi:10.1186/1475-2875-13-179)

## Slide 1
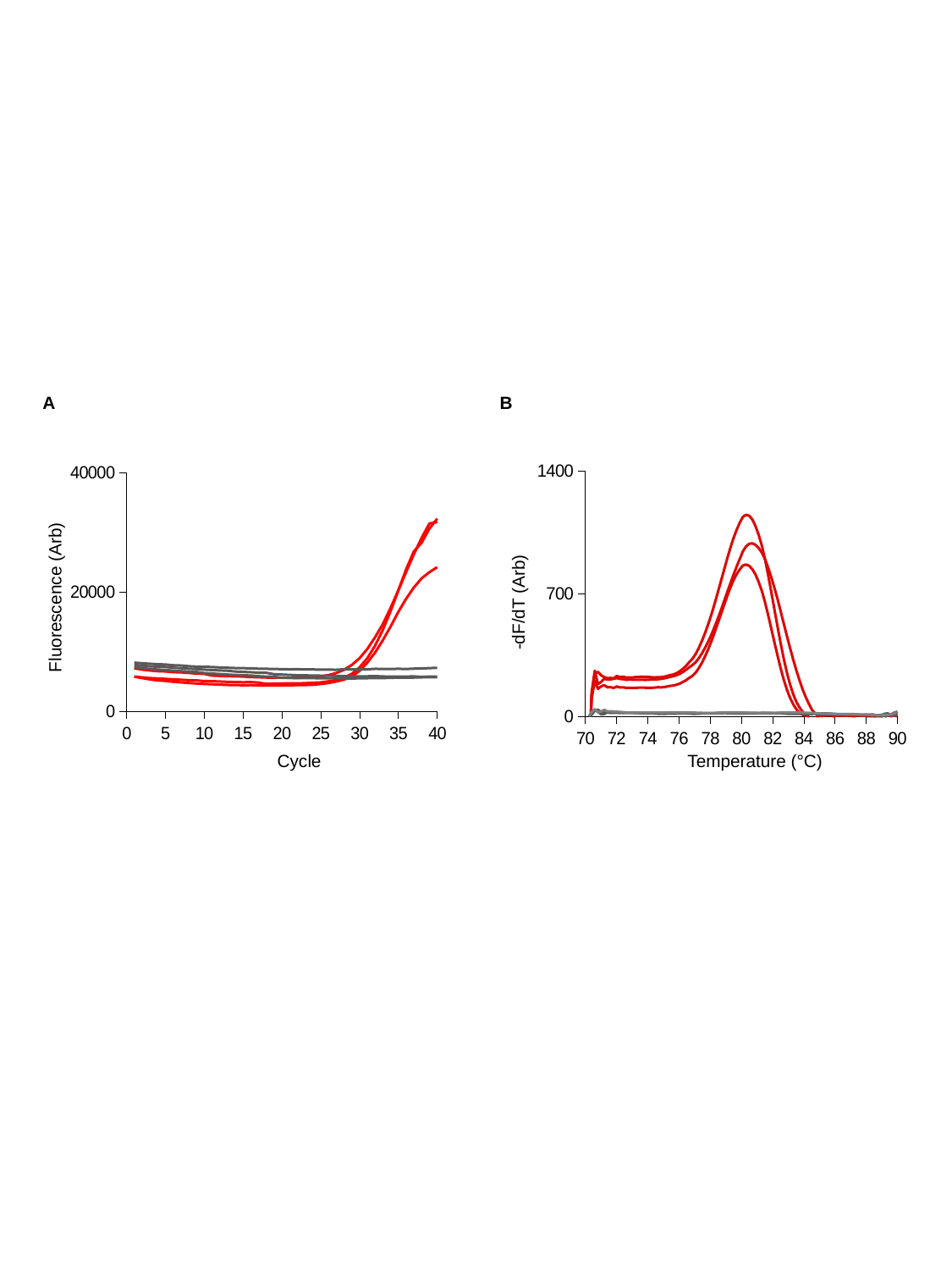

A
B
### Chart
| Category | | | | | | |
|---|---|---|---|---|---|---|
### Chart
| Category | | | | | | |
|---|---|---|---|---|---|---|Fluorescence (Arb)
-dF/dT (Arb)
Cycle
Temperature (°C)

Supplement: Additional file 2 — Hydrogel plastic chip controls. (A) PCR and (B) MCA curves for negative (gray) and positive (red) controls. [file 1475-2875-13-179-S2.pptx]
